# First-Principles Investigation of Interfacial Bonding, Stability, and Electronic Properties at the Fe(111)/Ti_3_SiC_2_(0001) Interface

**DOI:** 10.3390/nano16110647

**Published:** 2026-05-22

**Authors:** Xiangdong Wang, Wentao Li, Zhiwen Peng, Xiaoyu Yang, Mingjie Wang

**Affiliations:** 1 China Civil Engineering Construction Corporation, Beijing 100038, China; 2 China Academy of Ordnance Sciences Ningbo Branch, Ningbo 315000, China; 3 Inner Mongolia Metallic Materials Research Institute, Baotou 014034, China; 4 School of Intelligent Manufacturing, Huanghuai University, Zhumadian 463000, China

**Keywords:** Fe(111)/Ti_3_SiC_2_(0001) interfaces, first principle, heterogeneous nucleation, interfacial energy

## Abstract

A systematic first-principles density functional theory (DFT) study was performed using the Perdew–Burke–Ernzerhof (PBE) generalized gradient approximation (GGA) functional combined with ultrasoft pseudopotentials (USPPs), as implemented in the CASTEP code. The PBE-GGA functional was chosen because it provides a well-balanced description of both metallic and covalent bonding characteristics at the Fe/Ti_3_SiC_2_ interface. To elucidate the interfacial bonding mechanisms and heterogeneous nucleation behavior of Ti_3_SiC_2_ particles in iron-based composites. The structural stability, work of adhesion, interfacial energy, and electronic properties of the Fe(111)/Ti_3_SiC_2_(0001) interface were comprehensively investigated. A total of eighteen interface models were constructed, encompassing six distinct Ti_3_SiC_2_(0001) terminations: C(TiC), C(TiSi), TiC(TiC), TiC(TiSi), TiSi, and Si, and three stacking sequences (OT, MT, and HCP). The results demonstrate that the C(TiC)-terminated interface with HCP stacking exhibits the highest work of adhesion (9.25 J·m^−2^) and the lowest interfacial energy, thus representing the most thermodynamically stable configuration. Analysis of the partial density of states (PDOS) and charge density difference reveals that this exceptional stability originates from strong covalent bonding between Fe 3d and C 2p orbitals at the interface, accompanied by pronounced charge accumulation in the interfacial region. Furthermore, the work of adhesion of this interface substantially exceeds that of the fcc-Fe/fcc-Fe melt interface, confirming the high potency of Ti_3_SiC_2_ particles as heterogeneous nucleation substrates for Fe grains. These findings provide an atomistic framework for understanding the enhanced nucleation and robust interfacial cohesion observed in Fe/Ti_3_SiC_2_ composite coatings, and offer theoretical guidance for the design of advanced iron-based MAX phase composites.

## 1. Introduction

Iron-based alloys and steels remain indispensable structural materials in heavy machinery, rail transportation, and mining equipment, owing to their excellent mechanical properties, widespread availability, and cost-effectiveness [1,2,3]. However, critical moving components such as shafts, bearings, and gears are frequently subjected to harsh operating conditions, including high loads, elevated rotational speeds, and high temperatures [4,5], which result in severe friction and progressive wear. This tribological degradation leads to surface spalling, dimensional deviations, and, ultimately, catastrophic equipment failure or safety incidents.

To address these tribological challenges, surface modification techniques—including thermal spraying, ion implantation, and laser cladding—have been extensively developed [6,7,8]. Among these, laser cladding is regarded as a highly promising surface strengthening method owing to its narrow heat-affected zone, controllable dilution ratio, and metallurgical bonding between the coating and the substrate [9]. This technique enables the precise deposition of wear- and corrosion-resistant alloy or composite coatings onto component surfaces, thereby significantly extending their service life. Among various material systems, composite coatings reinforced with ternary MAX phase ceramics (such as Ti_3_AlC_2_ and Ti_2_AlN) have attracted considerable attention in recent years [10,11]. MAX phase materials possess a unique layered hexagonal structure that combines the ductility and thermal conductivity of metals with the high stiffness, high melting point, and excellent high-temperature oxidation resistance of ceramics. Consequently, they can effectively inhibit crack propagation and mitigate oxidative wear, providing reliable surface protection for critical components [12].

Titanium silicon carbide (Ti_3_SiC_2_) is a quintessential MAX phase that exhibits a unique combination of self-lubricating capacity, high-temperature stability, and excellent machinability [13,14]. Its nanolaminated crystal structure, comprising alternating Ti–C slabs and weakly bonded Si atomic layers, facilitates facile basal-plane slip under shear, thereby imparting an intrinsically low coefficient of friction and outstanding wear resistance [15]. Recent experimental studies have successfully fabricated Fe/Ti_3_SiC_2_ composite coatings on 45# steel via high-speed laser cladding, systematically optimizing the process parameters and Ti_3_SiC_2_ content [16]. Microscopic and spectroscopic analyses indicate that the improved tribological performance originates from the formation of a protective tribofilm enriched in partially decomposed Ti_3_SiC_2_, Fe-Ti-C intermetallics, and lubricious oxides. Nevertheless, the underlying atomistic mechanisms governing the nucleation and adhesion of Ti_3_SiC_2_ particles on the Fe matrix remain largely unexplored from a theoretical perspective.

First-principles density functional theory (DFT) calculations have evolved into an indispensable tool for deciphering the intricate electronic and structural properties of heterophase interfaces that are difficult to resolve solely through experimental means [17,18,19]. By constructing atomically precise coherent interface models, DFT enables the quantification of the work of adhesion, interfacial energy, and charge redistribution, thereby providing a direct assessment of interfacial bond strength and stability [20,21]. Prior DFT investigations on metal/MAX phase and metal–ceramic boundaries, including Al_2_O_3_/Ti_3_SiC_2_ and Cu/Ti_3_SiC_2_ interfaces, have demonstrated that the interfacial termination and the nature of metal–ceramic orbital hybridization decisively dictate the adhesion characteristics [22,23]. However, a dedicated theoretical study focusing on the Fe(111)/Ti_3_SiC_2_(0001) interface—the most relevant system for Fe-based composite coatings—is currently lacking. While density functional theory provides fundamental insights into interfacial bonding and adhesion at the atomistic scale, it is increasingly recognized that integrating DFT-derived parameters into larger-scale simulation frameworks offers a powerful pathway to connect atomistic predictions with experimental observations. Recent studies have demonstrated the successful implementation of multi-scale approaches, wherein first-principles calculations furnish critical input parameters for subsequent molecular dynamics or phase-field simulations of interface evolution and crystal growth. For instance, Filho et al. employed DFT-informed parameters to investigate crystal growth and interfacial processes at larger scales, demonstrating good agreement with experimental findings [24]. Similarly, Machado Filho et al. utilized DFT results as feed parameters for nanoscale simulations, enabling direct comparison with experimental observations of interfacial behavior [25]. These works underscore the importance of high-quality first-principles calculations, such as those presented in the current study, as foundational inputs for predictive multi-scale modeling of metal-ceramic interfaces.

In the present work, we employ DFT calculations to investigate the structural stability, electronic properties, and adhesion of the Fe(111)/Ti_3_SiC_2_(0001) interface. A series of interface models with distinct terminations and stacking sequences are constructed to identify the most energetically favorable configuration. The work of adhesion and interfacial energy are computed to evaluate the intrinsic bond strength. Charge density differences and density of states analyses are employed to reveal the dominant orbital interactions. The overarching objective is to establish an atomistic framework that rationalizes the enhanced nucleation and robust cohesion observed experimentally in Fe/Ti_3_SiC_2_ laser-clad coatings, thereby offering predictive guidance for the compositional design of next-generation self-lubricating composites.

## 2. Computational Methods

**Figure 1:**
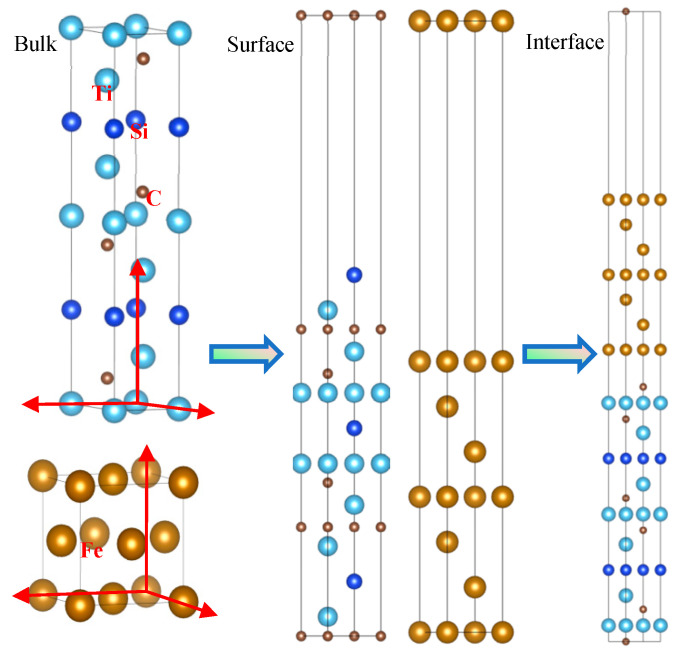
The crystal structure of bulk Ti_3_SiC_2_ and fcc-Fe; the surface of Ti_3_SiC_2_(0001) and Fe(111) slabs; and the interface of Ti_3_SiC_2_(0001)/Fe(111).

All first-principles calculations were performed using the Cambridge Sequential Total Energy Package (CASTEP) code [26,27]. Exchange–correlation contributions were treated with the Perdew–Burke–Ernzerhof (PBE) functional within the generalized gradient approximation (GGA) [28]. Electron–ion interactions were described by ultrasoft pseudopotentials (USPPs) implemented in reciprocal space [29,30]. A plane-wave kinetic energy cutoff of 500 eV was employed for all calculations. The following valence electron configurations were adopted: Ti (3d^3^4s^1^), Si (3s^2^3p^2^), Fe (3d^7^4s^1^), and C (2s^2^2p^2^) [31]. Brillouin-zone integrations were performed using the Monkhorst–Pack scheme with k-point meshes of 12 × 12 × 12 for the Fe and Ti_3_SiC_2_ bulk phases, and 12 × 12 × 1 for all surface and interfacial structures (Figure 1). Structural relaxation was carried out using Broyden–Fletcher–Goldfarb–Shanno (BFGS) minimization algorithm to identify the lowest-energy interfacial configuration [32]. Kohn–Sham equations were solved self-consistently until the electronic energy converged [33]. During optimization, the lattice parameters were held constant while atomic coordinates were allowed to relax. Convergence criteria were defined as follows: an energy change per atom of less than 1.0 × 10^−6^ eV, a maximum Hellmann–Feynman force component of 0.03 eV·Å^−1^, a maximum atomic displacement of 0.001 Å, and a maximum stress component of 0.02 GPa.

The in-plane supercell dimensions were determined by matching a (1 × 1) hexagonal surface cell of Fe(111) with a (1 × 1) surface cell of Ti_3_SiC_2_(0001), yielding an average in-plane lattice constant of 5.11 Å and a lattice mismatch below 5%. The thicknesses of the Fe(111) and Ti_3_SiC_2_(0001) slabs were converged through systematic surface energy tests, resulting in 7 and 13 atomic layers, respectively. A vacuum layer of 15 Å was found sufficient to avoid artificial periodic interactions. k-point convergence tests confirmed that a 12 × 12 × 1 Monkhorst–Pack grid ensures energy convergence to within 2 meV per atom for all interface models.

It should be noted that while bcc-Fe is in the thermodynamically stable phase at room temperature, the fcc structure was adopted in this work because laser cladding involves rapid melting and solidification processes, where the high-temperature γ-Fe (fcc) phase forms at the melt pool interface. Moreover, previous DFT studies on Fe-based coatings have successfully employed the fcc-Fe structure to model interfacial phenomena at elevated temperatures. The calculated lattice constant for fcc-Fe is a = 3.655 Å, while those for Ti_3_SiC_2_ are a = b = 3.070 Å and c = 17.693 Å, which is in reasonable agreement with the experimental value [34,35] and previous computational results [36,37], strongly corroborating the findings and confirming the kinetic stability of Ti_3_SiC_2_.

## 3. Results and Discussion

### 3.1. Surface Properties of Ti_3_SiC_2_

**Figure 2:**
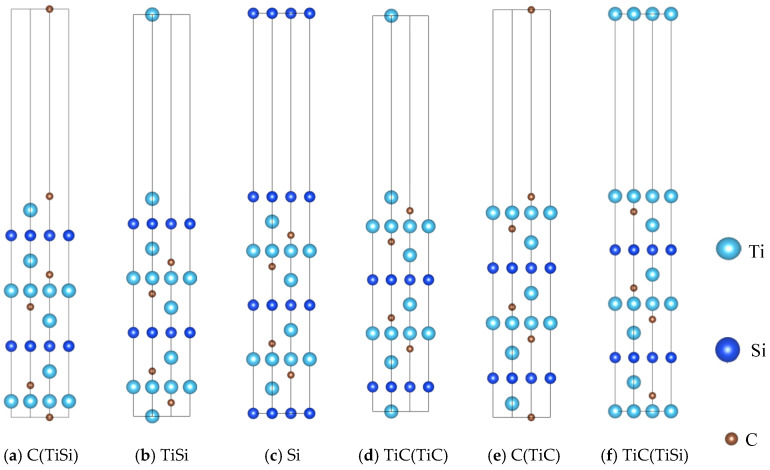
Schematic diagram of Ti_3_SiC_2_(0001) surface of different terminations.

To systematically investigate the Fe/Ti_3_SiC_2_ interface, the intrinsic properties of the Fe(111) and Ti_3_SiC_2_(0001) surfaces were first examined. Based on previous research [38], six distinct surface termination types and 18 surface models were constructed; the corresponding terminations of the Ti_3_SiC_2_(0001) surfaces are illustrated in Figure 2. The most stable surface model from each termination type was selected based on convergence tests of surface energy and interlayer spacing. The test results showed that when the number of surface layers reached 13, the Ti_3_SiC_2_(0001) surfaces that were terminated by TiC, C, C(TiC), C(TiSi), TiSi, TiC(TiSi), and Si all gradually converged. Therefore, due to the different stacking methods between Ti_3_SiC_2_(0001) thin films with different end faces, 18 Ti_3_SiC_2_(0001)/Fe(111) interface models were constructed.

To quantitatively assess the effect of termination chemistry on the structural stability of the Ti_3_SiC_2_(0001) surface, the surface energies of all terminations were computed and compared. This energy value serves as a direct indicator of the relative stability among various surface configurations: a smaller surface energy implies a more thermodynamically preferred terminal arrangement. Within the symmetric slab model adopted in the present work, the surface energy is obtainable via the formula given in the cited literature [20].

$$\sigma =\frac{1}{2A}\left(E_{slab}-N_{Ti}\mu_{Ti}^{slab}-N_{Si}\mu_{Si}^{slab}-N_{C}\mu_{C}^{slab}-PV-TV\right)$$

In the present calculation, E_slab_ denotes the total energy of a fully relaxed Ti_3_SiC_2_ surface. The symbols $\mu_{\mathrm{Ti}}^{slab}$, $\mu_{\mathrm{Si}}^{slab}$, and $\mu_{C}^{slab}$ represent the energies of Ti, Si, and C atoms within the surface slab, respectively. Meanwhile, N_Ti_, N_Si_, and N_C_ give the corresponding atomic counts of Ti, Si, and C in the same surface model. Surface area is denoted by A. Any contributions from PV and TS are negligible, as the simulation is carried out at 0 K under ground-state conditions. Within this system, the chemical potential of an individual atom is equal to that of the bulk phase. Consequently, the chemical potential together with the surface energy for bulk Ti_3_SiC_2_ can be expressed as:

$$\mu_{{Ti}_{3}SiC_{2}}^{Bulk}={3\mu }_{Ti}+\mu_{Si}+{2\mu }_{C}$$

$$\mu_{{Ti}_{3}SiC_{2}}^{Bulk}=3\mu_{Ti}^{Bulk}+\mu_{Si}^{Bulk}+2\mu_{C}^{Bulk}+∆H_{f}$$

Therefore, the equation surface energy can be finally written as:

$$\sigma =\frac{1}{2A}\left(E_{slab}-N_{Si}\mu_{{Cr}_{2}AlC}^{Bulk}+\left(3{N_{Si}-N}_{Ti}\right)(∆\mu_{Ti}+\mu_{Ti}^{Bulk})+\left({{2N}_{Si}-N}_{C}\right)\left(∆\mu_{C}+\mu_{Cl}^{Bulk}\right)\right)$$

$$∆\mu_{Ti}=\mu_{Ti}-\mu_{Ti}^{Bulk}\;\;\;\;\;\;\;\;∆\mu_{C}=\mu_{C}-\mu_{C}^{Bulk}$$

For a thermodynamically stable Ti_3_SiC_2_ surface, the chemical potential of each atomic species must not exceed that of its corresponding elemental reference state; otherwise, the slab would become unstable. Therefore, the inequalities $\mu_{Ti}$ ≤ $\mu_{Ti}^{Bulk}$, $\mu_{Si}$ ≤ $\mu_{Si}^{Bulk}$, and $\mu_{C}$ ≤ $\mu_{C}^{Bulk}$ constitute necessary preconditions for forming a stable surface structure.

According to above equations:

$$3\mu_{\mathrm{Ti}}+\mu_{\mathrm{Si}}+2\mu_{C}=3\mu_{\mathrm{Ti}}^{Bulk}+\mu_{\mathrm{Si}}^{Bulk}+2\mu_{C}^{Bulk}+\Delta H_{f}$$

Thus,

$$3\Delta \mu_{\mathrm{Ti}}+\Delta \mu_{\mathrm{Si}}+2\Delta \mu_{C}=\Delta H_{f}$$

$$\Delta H_{f}\leq 3\Delta \mu_{\mathrm{Ti}}+2\Delta \mu_{C}\leq 0$$

The formation enthalpy of bulk Ti_3_SiC_2_ is −5.29 eV in previous research [23]. Thus,

$$-5.29\leq 3\Delta \mu_{\mathrm{Ti}}+2\Delta \mu_{C}\leq 0$$

**Figure 3:**
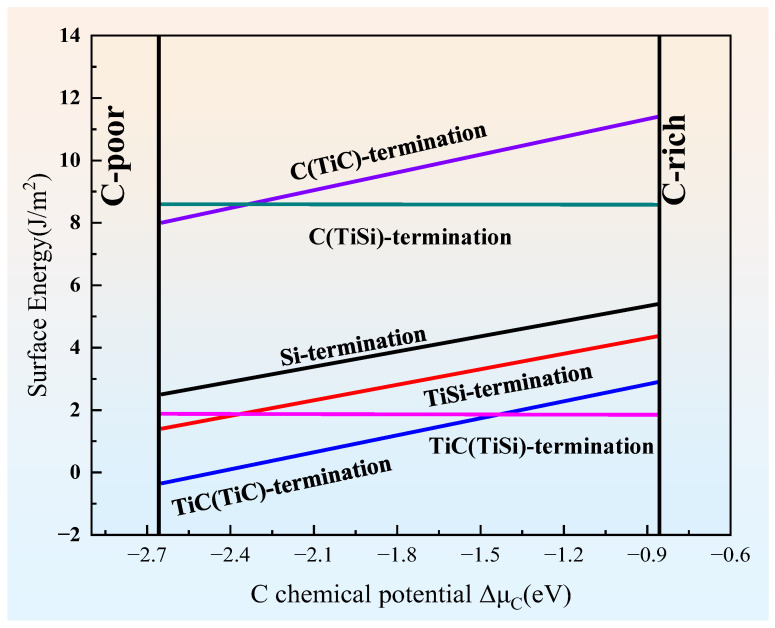
The surface energy of Ti_3_SiC_2_(0001) as a function of $\Delta \mu_{C}$ when $\Delta \mu_{\mathrm{Ti}}=0$.

For the Ti_3_SiC_2_(0001) surface, the surface energy of Ti_3_SiC_2_(0001) slabs varies with changes of $\Delta \mu_{C}$, and $\Delta \mu_{\mathrm{Ti}}=-1.763-\frac{2}{3}\Delta \mu_{C}$. As shown in Figure 3, a higher C value indicates a carbon-rich state, whereas a lower C value indicates a carbon-poor state. Figure 3 reveals a linear dependence of the surface energy of the polar Ti_3_SiC_2_(0001) surface on the carbon chemical potential $\Delta \mu_{C}$. By taking specific terminations as examples, such as the C(TiC)-, TiC(TiC)-, TiSi-, and Si-terminated surfaces, their surface energies grow linearly with increasing $\Delta \mu_{C}$. In contrast, the C(TiSi) and TiC(TiSi) terminations show virtually no change in surface energy as $\Delta \mu_{C}$ rises. Under carbon-rich conditions, the surface energies of the TiSi, Si, C(TiSi), and TiC(TiSi) terminations become comparable to each other, implying that these four types can coexist. When $\Delta \mu_{C}$ is low, the TiC(TiC) termination possesses the smallest surface energy, therefore being thermodynamically most stable. However, as $\Delta \mu_{C}$ increases, the surface energy of this termination gradually rises; once $\Delta \mu_{C}$ exceeds −1.50 eV, the TiC(TiSi) termination takes over as the most stable configuration. Notably, over the whole $\Delta \mu_{C}$ range being considered, the C(TiC) and C(TiSi) terminations always exhibit higher surface energies than all other terminations, indicating that they are the least stable ones.

### 3.2. Interfacial Adhesion and Stability of the Fe(111)/Ti_3_SiC_2_(0001) Interface

**Figure 4:**
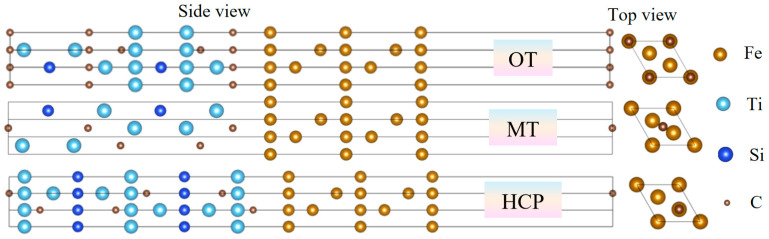
Schematic diagram of three classical interface stacking methods from top and side views.

Based on the convergence test outcomes from the preceding section, interface models were constructed by stacking variously terminated Ti_3_SiC_2_(0001) surfaces onto a seven-layer Fe(111) slab. A vacuum layer of 15 Å thickness was introduced atop the resulting supercell to eliminate spurious periodicity effects on interatomic interactions. Four distinct stacking arrangements were considered for the Fe(111)/Ti_3_SiC_2_(0001) system, depending on the relative alignment of atoms between the two surfaces. This procedure generated 18 interfacial configurations, which were subsequently subjected to detailed characterization. Three high-symmetry stacking sites are defined as follows (Figure 4): OT denotes the position where a Ti_3_SiC_2_(0001) surface atom lies directly above an Fe atom; MT corresponds to the hollow site at the center of four neighboring Fe atoms; and HCP represents the site at the center of three adjacent Fe atoms.

Therefore, according to the symmetry of the crystal structure, the Ti_3_SiC_2_(0001) surface corresponds to six different terminal structures, while the Fe(111) surface has only one terminal structure. Therefore, depending on the stacking mode, we can construct 18 different interfaces: OT-stacked, MT-stacked, and HCP-stacked C(TiSi)-Ti_3_SiC_2_(0001)/Fe(111) interfaces; OT-stacked, MT-stacked, and HCP-stacked C(TiC)-Ti_3_SiC_2_(0001)/Fe(111) interfaces; OT-stacked, MT-stacked, and HCP-stacked TiC(TiSi)-Ti_3_SiC_2_(0001)/Fe(111) interfaces; OT-stacked, MT-stacked, and HCP-stacked TiC(TiC)-Ti_3_SiC_2_(0001)/Fe(111) interfaces; OT-stacked, MT-stacked, and HCP-stacked TiSi-Ti_3_SiC_2_(0001)/Fe(111) interfaces; and OT-stacked, MT-stacked, and HCP-stacked Si-Ti_3_SiC_2_(0001)/Fe(111) interfaces.

The work of adhesion (W_ad_), defined as the energy required to separate a unit area of interface into two free surfaces, is adopted as the standard metric to quantify interfacial bonding strength. This area-normalized quantity enables direct comparison across different interface configurations and with the literature values for other metal/ceramic systems. The work of adhesion (W_ad_) and interfacial energy (γ_int_) jointly characterize the bonding properties of the Fe(111)/Ti_3_SiC_2_(0001) interface. Specifically, W_ad_ is defined as the minimum work required to separate the interface into two free surfaces; this quantity is positively related to the interfacial bond strength. In contrast, γ_int_ reflects the thermodynamic stability of the interface. It represents the energy released upon forming a unit area of the interface, and a lower γ_int_ corresponds to higher stability. The corresponding calculation formulas are given as follows [39]:

$$Wad=\frac{E_{\mathrm{Fe}}+E_{Ti_{3}SiC_{2}}-E_{Fe/Ti_{3}SiC_{2}}}{A}$$

$$\gamma_{\mathrm{int}}=\gamma_{\mathrm{Fe}}+\gamma_{Ti_{3}SiC_{2}}-W_{ad}$$

where $E_{\mathrm{Fe}}$, $E_{{\mathrm{Ti}}_{3}SiC_{2}}$, and $E_{{\mathrm{Fe}/\mathrm{Ti}}_{3}SiC_{2}}$ are the total energies of Fe(111) surface, Ti_3_SiC_2_(0001) surface, and Fe(111)/Ti_3_SiC_2_(0001) interface, respectively, while A represents the interface area. $\gamma_{\mathrm{Fe}}$ and $\gamma_{{\mathrm{Ti}}_{3}{\mathrm{SiC}}_{2}}$ are the surface energies of Fe(111) and Ti_3_SiC_2_(0001) surfaces, respectively.

**Figure 5:**
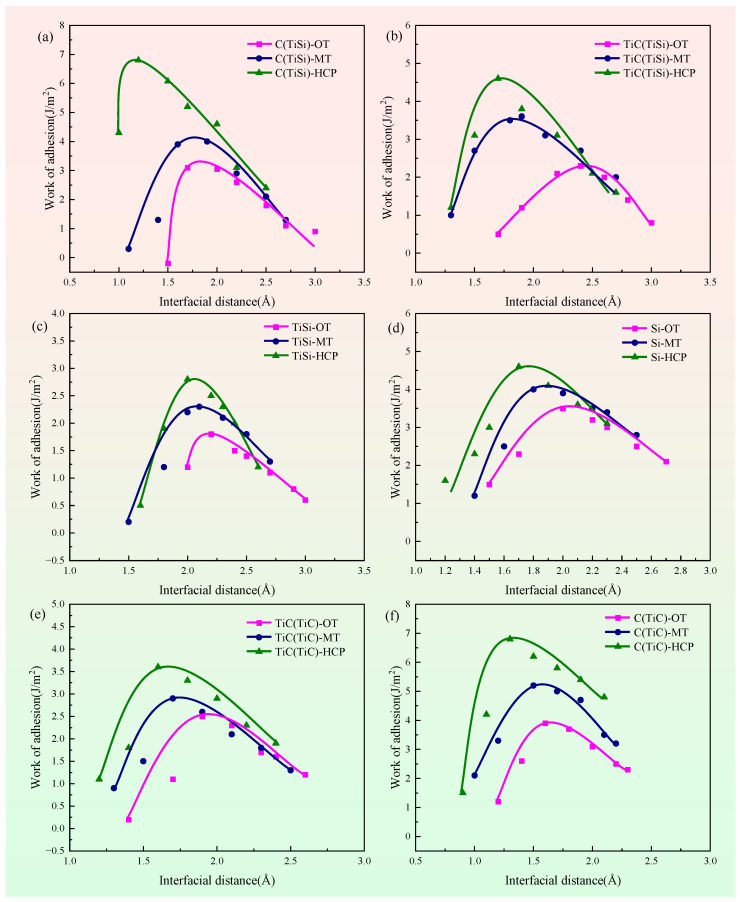
(**a**–**f**) Adhesion energy as a function of interfacial separation distance for the Fe(111)/Ti_3_SiC_2_(0001) interfaces with different terminations and stacking sequences, calculated using the Universal Binding Energy Relation (UBER) approach.

To reduce computational expense, the equilibrium interfacial spacing is determined from all interface models via the Universal Binding Energy Relation (UBER) method, prior to fully relaxing the model structures. The detailed workflow consists of three steps. Firstly, we construct a series of atomic configurations featuring varying initial interface separations (denoted as d_0_). We keep atomic positions fixed and compute the total energy for each configuration. Secondly, we employ the adhesion energy formula to evaluate the work of adhesion (W_ad_) associated with every separation distance. Lastly, we generate a plot showing how the adhesion energy changes with interfacial distance (presented in Figure 5). Finally, we identify the equilibrium distance by applying the energy minimization criterion.

As illustrated in Figure 5, the interfacial adsorption behavior is jointly governed by the stacking geometry and the separation between the two surfaces. Relative to the OT and MT stacking configurations, the HCP-stacked interface yields markedly higher adsorption energies for an Fe-terminated Fe(111) surface paired with a Ti_3_SiC_2_(0001) surface bearing any of the following terminations: TiC(TiC), TiSi, C(TiSi), C(TiC), TiC(TiSi), or Si. Finally, the computed interfacial work of adhesion for the three stacking patterns rank as HCP > MT > OT. For combinations formed by the TiC(TiC), TiSi, C(TiSi), C(TiC), TiC(TiSi), and Si-terminated Ti_3_SiC_2_(0001) surfaces together with the Fe(111) surface, the sequence of work of adhesion remains identical. In contrast, for the six interfacial terminations examined, the optimal interfacial separation exhibits the opposite trend to that of the adsorption energies, decreasing in the order OT > MT > HCP. In addition, as seen in Figure 5, the highest adsorption energy occurs for the interface formed by C(TiC)-terminated Ti_3_SiC_2_(0001) and Fe(111), which implies that it is the most thermodynamically stable. Among the six optimal interfacial configurations examined, the adsorption energies decrease in the order of C(TiC) > C(TiSi) > TiC(TiC) > Si > TiC(TiSi) > TiSi, whereas the corresponding optimal interfacial separations follow the opposite trend: OT > MT > HCP. After the optimal interfacial spacing was established, each interface model was structurally relaxed at that separation. The final calculations yielded the optimal spacing values before and after relaxation, together with the corresponding adhesion energies; the detailed results are compiled in Table 1.

Two principal trends emerge from the data compiled in Table 1. First, for interfaces sharing the same termination, structural relaxation substantially enhances the work of adhesion while exerting only a marginal effect on the equilibrium interfacial spacing. Second, the work of adhesion varies considerably across different terminations, indicating that the termination chemistry is the dominant factor governing interfacial stability. By contrast, the influence of stacking geometry on the adsorption energy is comparatively modest. The computed results agree well with those reported in reference [40], confirming the reliability of the computational methodology. The HCP-stacked C(TiC)-Ti_3_SiC_2_(0001)/Fe(111) interface exhibits a work of adhesion of 9.25 J·m^−2^, the highest among all modeled configurations, indicating its optimal thermodynamic stability. Consequently, the six most stable HCP-stacked interfaces were selected for subsequent electronic structure calculations to elucidate the reinforcement mechanisms of the Ti_3_SiC_2_(0001)/Fe(111) interface.

### 3.3. Electronic Structure of the Fe(111)/Ti_3_SiC_2_(0001) Interface

To elucidate the charge distribution across the interface and its influence on interfacial bonding, six HCP-stacked configurations of the Ti_3_SiC_2_(0001)/Fe(111) interface were examined. Particular emphasis was placed on three quantities: the total electron density, the charge density difference, and the site-resolved partial density of states (PDOS).

**Figure 6:**
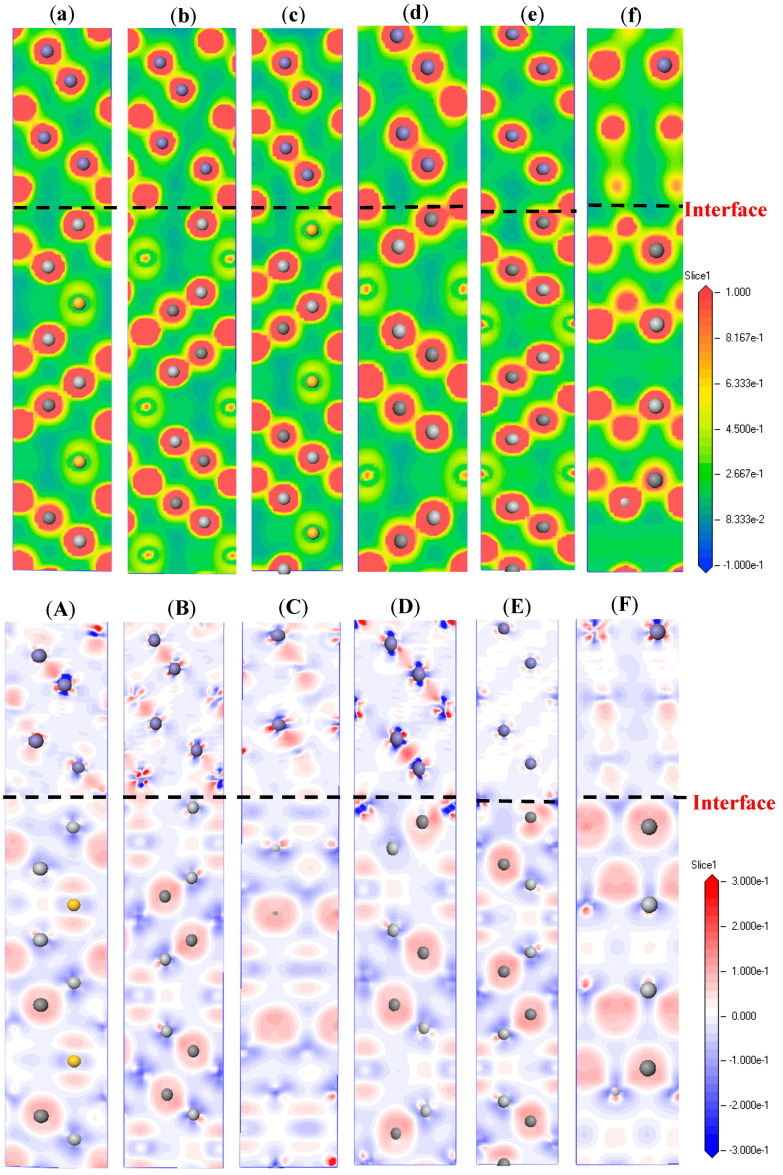
The charge density distribution and charge density differences for six different interface models: (**a**,**A**) HCP-stacked TiC(TiSi)-Ti_3_SiC_2_(0001)/Fe(111) interface; (**b**,**B**) HCP-stacked TiSi-Ti_3_SiC_2_(0001)/Fe(111) interface; (**c**,**C**) HCP-stacked Si-Ti_3_SiC_2_(0001)/Fe(111) interface; (**d**,**D**) HCP-stacked (TiSi)-Ti_3_SiC_2_(0001)/Fe(111) interface; (**e**,**E**) HCP-stacked TiC(TiC)-Ti_3_SiC_2_(0001)/Fe(111) interface; and (**f**,**F**) HCP-stacked C(TiC)-Ti_3_SiC_2_(0001)/Fe(111) interface.

As shown in Figure 6, the electron density distribution and its differential vary across the six interfacial models. For interfaces involving identical atomic species, pronounced charge depletion is observed, with the depleted electrons migrating toward the interfacial region. This charge redistribution is characteristic of metallic bonding. Conversely, at interfaces between dissimilar atomic species, substantial charge accumulation is observed, facilitating the formation of directional, saturated covalent bonds. These contrasting charge redistribution patterns underscore the intrinsic differences in chemical bonding across the various interface configurations. Figure 6 further reveals a marked disparity in charge transfer between interfacial Fe atoms and those in the bulk interior. Fe atoms in the inner layers exhibit modest charge dissipation with only minor lattice distortions induced by interatomic interactions, whereas interfacial Fe atoms display pronounced charge polarization. The spatial extent of Fe charge depletion originates in the immediate vicinity of the interface and extends into the adjacent Ti_3_SiC_2_(0001) surface layers.

The charge density maps reveal that the elevated electronegativity of Ti and C facilitates partial charge transfer from interfacial Fe atoms, generating substantial charge accumulation within the interfacial region. Among the various terminations, the C(TiC) interface exhibits the most pronounced Fe-C charge transfer. The distinct relative atomic arrangements at different terminations inherently give rise to divergent charge redistribution characteristics. Notably, compared to the remaining five configurations, the C(TiC)-Ti_3_SiC_2_(0001)/Fe(111) interface exhibits significantly enhanced charge depletion and accumulation, concomitant with a markedly reduced interfacial spacing.

**Figure 7:**
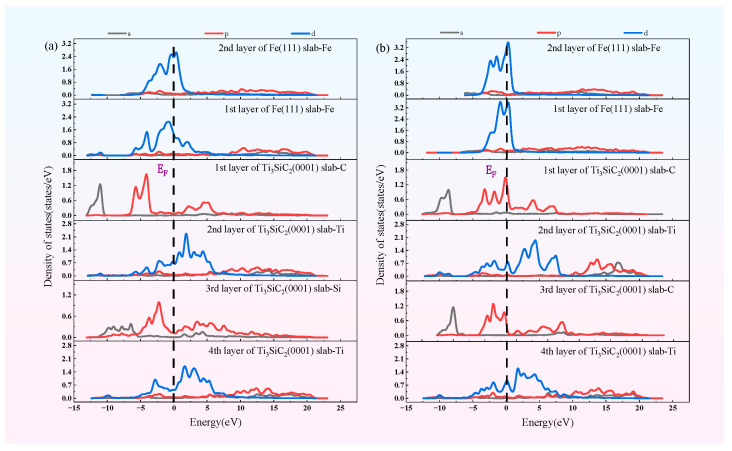

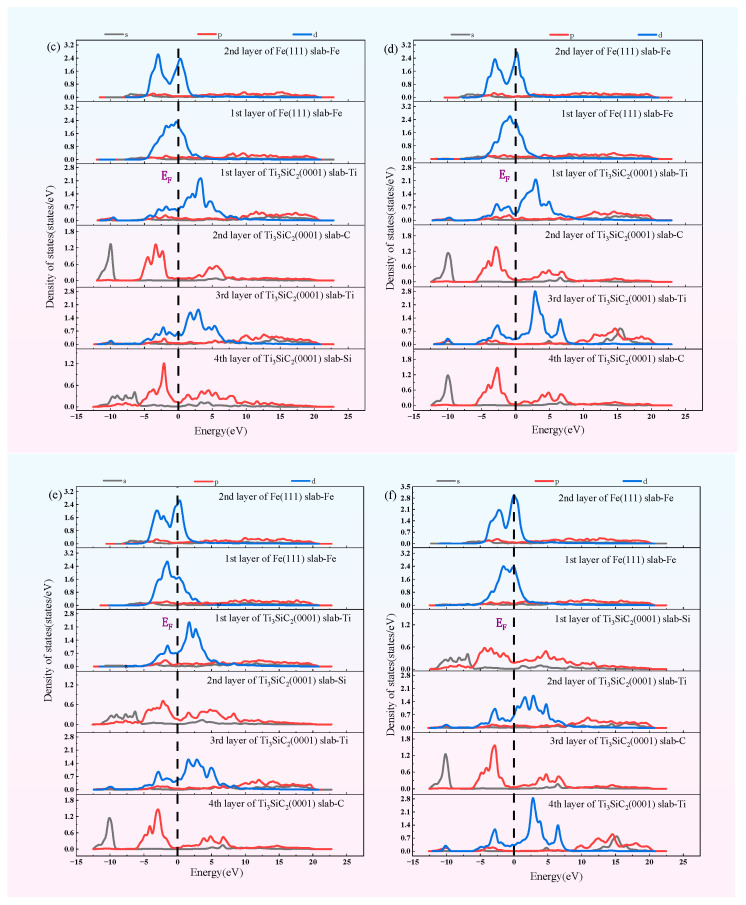
The partial density of states for six different interface models: (**a**) HCP-stacked C(TiSi)-Ti_3_SiC_2_(0001)/Fe(111) interface; (**b**) HCP-stacked C(TiC)-Ti_3_SiC_2_(0001)/Fe(111) interface; (**c**) HCP-stacked (TiSi)-Ti_3_SiC_2_(0001)/Fe(111) interface; (**d**) HCP-stacked TiC(TiC)-Ti_3_SiC_2_(0001)/Fe(111) interface; (**e**) HCP-stacked TiSi-Ti_3_SiC_2_(0001)/Fe(111) interface; and (**f**) HCP-stacked Si-Ti_3_SiC_2_(0001)/Fe(111) interface.

Further insight into the bonding character between dissimilar atoms and underlying mechanisms can be obtained by examining the electronic states at the interface. To this end, the partial density of states (PDOS) for the s, p, and d orbitals of atoms situated near the interface were calculated, permitting an assessment of the contribution of each orbital to interfacial bonding. The PDOS distributions for the six interface models are presented in Figure 7, enabling a direct comparison of the electronic structural characteristics across the different configurations.

Examination of the PDOS profiles for atoms at different positions (Figure 7) reveals that interfacial Fe atoms exhibit polarization characteristics distinct from those of bulk-like interior Fe atoms, manifested by more pronounced oscillatory features and suppressed PDOS magnitudes at the Fermi level. These features are indicative of enhanced nonmetallic character in the interfacial Fe atoms. For the C(TiC)-Ti_3_SiC_2_(0001)/Fe(111) interface (Figure 7f), the PDOS curves of both C and Fe are characterized by intense peaks and broad distributions, implying significant electron localization and the formation of robust covalent bonds at the interface. A pronounced hybridization peak is observed near −1.5 eV, originating primarily from the Fe 3d and C 2p orbitals. In comparison, the C(TiSi)-Ti_3_SiC_2_(0001)/Fe(111) interface (Figure 7d) exhibits a hybridization peak near −4.5 eV of considerably lower intensity, along with reduced PDOS values at the Fermi level, suggesting inferior interfacial stability relative to the C(TiC) interface. For the remaining four terminations (Figure 7c–f), the PDOS curves of interfacial Fe, Ti, and Si are flatter than those in the bulk-like regions, with lower PDOS values at the Fermi level. This indicates that, although some charge depletion occurs at interfacial Fe atoms, no pronounced hybridization peaks are present; consequently, these interfaces retain a predominantly metallic character. Collectively, these electronic structure analyses confirm that the C(TiC)-Ti_3_SiC_2_(0001)/Fe(111) interface exhibits the strongest bonding, consistent with the adhesion work, interfacial energy, and interfacial spacing analyses.

To further investigate the chemical bonding at the Ti_3_SiC_2_(0001)/Fe(111) interface, we compared the charge population, bond population, and bond length of three types of atoms near the C(TiC)-terminated and C(TiSi)-terminated interfaces with HCP stacking. The results are presented in Table 2 and Table 3. As shown in the tables, the Fe-C bond at the C(TiC)-terminated interface has a bond population of 1.27 and a bond length of 1.87 Å, while at the C(TiSi)-terminated interface, the Fe-C bond exhibits a bond population of 1.18 and a bond length of 1.96 Å. These data indicate that the Fe-C covalent bond at the C(TiC)-terminated Fe(111)/Ti_3_SiC_2_(0001) interface is stronger in covalency and has a shorter bond length, suggesting that interface Fe atoms transfer electrons to C atoms, resulting in a more stable interface.

To evaluate the heterogeneous nucleation behavior of Ti_3_SiC_2_ particles in iron-based alloys, the work of adhesion of the most stable Fe(111)/Ti_3_SiC_2_(0001) interfaces was compared with the cohesive energy of molten Fe. Although the interfacial properties reported herein were computed at 0 K using CASTEP, previous studies have demonstrated that such first-principles results correlate well with high-temperature experimental data for solid–solid and solid–liquid interfaces [41,42]. The heterogeneous nucleation analysis reveals that, among the six interface configurations examined, the HCP-stacked C(TiC)-Ti_3_SiC_2_(0001)/Fe(111) interface is the most favorable for heterogeneous nucleation of Fe. This enhanced potency is attributed to its highest work of adhesion, strongest interfacial bonding, and most favorable electronic structure. Moreover, the work of adhesion of this interface (9.25 J·m^−2^) substantially exceeds that of the fcc-Fe/fcc-Fe melt interface (6.36 J·m^−2^) [43], underscoring the potent grain-refining effect of Ti_3_SiC_2_ particles in iron-based alloys. This theoretical prediction is consistent with available experimental observations.

## 4. Conclusions

In this work, the interfacial properties, electronic structure, and heterogeneous nucleation mechanism of the Fe(111)/Ti_3_SiC_2_(0001) interface were systematically investigated by means of first-principles density functional theory calculations. The principal conclusions are as follows:

(1) Based on surface energy convergence tests for six distinct Ti_3_SiC_2_(0001) terminations, a total of eighteen Fe(111)/Ti_3_SiC_2_(0001) interface models were constructed, incorporating OT, MT, and HCP stacking sequences. Among these, the C(TiC)-terminated interface adopting the HCP stacking configuration was identified as the most thermodynamically stable, exhibiting the highest work of adhesion (9.25 J·m^−2^) and the lowest interfacial energy.
(2) Both the work of adhesion and the interfacial energy exhibit greater sensitivities to termination type than to stacking sequence. Across the three stacking arrangements, HCP stacking consistently yields the strongest interfacial binding, followed by MT and OT.
(3) Electronic structure analyses demonstrate that the exceptional stability of the C(TiC)-terminated HCP interface originates from the formation of strong Fe–C covalent bonds, as evidenced by pronounced hybridization between Fe 3d and C 2p orbitals in the vicinity of the Fermi level. Charge density difference maps further corroborate substantial electron transfer from interfacial Fe atoms to C atoms, thereby reinforcing the interfacial bonding.
(4) The calculated work of adhesion for the C(TiC)-terminated Fe(111)/Ti_3_SiC_2_(0001) interface substantially exceeds that of the fcc-Fe/fcc-Fe melt interface, underscoring the potent heterogeneous nucleation catalytic effect of Ti_3_SiC_2_ particles on Fe grains. This theoretical prediction is in good agreement with available experimental observations and provides valuable insight for the rational design of advanced iron-based composite coatings.

It should be noted that the present study is based on static DFT calculations at 0 K. While this approach provides fundamental insights into interfacial adhesion, bonding mechanisms, and electronic structures, finite-temperature effects such as thermal expansion, vibrational entropy contributions, and potential interface reconstructions at elevated temperatures are not explicitly considered. Ab initio molecular dynamics simulations would be valuable for investigating the thermal stability and high-temperature behavior of the Fe(111)/Ti_3_SiC_2_(0001) interface, including potential atomic diffusion and disordering effects. Such investigations represent an important direction for our future work and would complement the present 0 K ground-state analysis.

## Figures and Tables

**Table 1:** Interface spacing, work of adhesion, and interface energy before and after relaxation of 36 Ti_3_SiC_2_(0001)/Fe(111) interfaces.

Termination		Stacking	Unrelaxed		Relaxed		
Fe	Ti_3_SiC_2_		d_0_/Å	W_ad_ (J/m^2^)	d_0_/Å	W_ad_ (J/m^2^)	γ_int_ (J/m^2^)
Fe	C(TiC)	OT	1.6	3.92	1.5	5.54	1.15
		MT	1.5	5.19	1.3	7.13	0.95
		HCP	1.3	7.18	1.2	9.25	−1.03
	C(TiSi)	OT	2.0	3.15	1.8	4.56	1.65
		MT	1.9	4.05	1.8	5.74	0.13
		HCP	1.2	6.85	1.2	8.28	−0.95
	TiC(TiSi)	OT	2.4	2.35	2.3	3.52	5.09
		MT	1.9	3.61	1.8	3.96	3.00
		HCP	1.7	4.69	1.5	6.85	−0.12
	TiC(TiC)	OT	1.9	2.56	1.8	3.86	2.21
		MT	1.7	2.94	1.7	5.14	1.05
		HCP	1.6	3.67	1.5	5.65	1.23
	TiSi	OT	2.2	1.82	2.1	2.87	3.64
		MT	2.1	2.35	2.0	3.95	2.51
		HCP	2.0	2.89	1.9	4.18	1.32
	Si	OT	2.0	3.51	2.0	4.38	1.38
		MT	1.9	4.06	1.8	5.42	1.12
		HCP	1.8	4.32	1.7	6.23	−0.07

**Table 2:** Mulliken charges on representative atoms in the Ti_3_SiC_2_(0001)/Fe(111) interface.

Termination	Atom	s	p	d	f	Total	Charge(e)
C(TiC)	Fe	0.3	0.58	6.8	0	7.68	0.32
	C	1.47	3.2	0	0	4.67	−0.67
	Ti	2.18	6.73	2.74	0	11.66	0.34
C(TiSi)	Fe	0.35	0.65	6.74	0	7.74	0.26
	C	1.51	3.07	0	0	4.57	−0.57
	Ti	2.11	6.64	2.65	0	11.4	0.6

**Table 3:** The bond lengths and population of the Ti_3_SiC_2_(0001)/Fe(111) interface.

Termination	Bond	Population	Length (Å)
C(TiC)	C-Fe	1.27	1.87
	C-Ti	0.65	2.16
C(TiSi)	C-Fe	1.18	1.96
	C-Ti	0.68	2.29

## Data Availability

The data presented in this study are available on request from the corresponding author. The data are not publicly available because some or all data, models, and code generated or used during the study are proprietary or confidential in nature.
